# Metabolic and Hormonal Changes After Laparoscopic Roux-en-Y Gastric Bypass and Sleeve Gastrectomy: a Randomized, Prospective Trial

**DOI:** 10.1007/s11695-012-0622-3

**Published:** 2012-02-22

**Authors:** Ralph Peterli, Robert E. Steinert, Bettina Woelnerhanssen, Thomas Peters, Caroline Christoffel-Courtin, Markus Gass, Beatrice Kern, Markus von Fluee, Christoph Beglinger

**Affiliations:** 1 Department of Surgery, St. Claraspital, 4016 Basel, Switzerland; 2 Clinical Research Center, Department of Biomedicine, University Hospital, 4031 Basel, Switzerland; 3 Department of Surgery, University Hospital, 4031 Basel, Switzerland; 4 Department of Medicine, St. Claraspital, 4016 Basel, Switzerland; 5 Department of Gastroenterology, University Hospital, 4031 Basel, Switzerland

**Keywords:** Metabolic surgery, Glycemic control, Laparoscopic Roux-en-Y gastric bypass, Laparoscopic sleeve gastrectomy, Gut hormones, Cholecystokinin (CCK), Ghrelin, Glucagon-like peptide 1 (GLP-1), Peptide YY (PYY)

## Abstract

**Background:**

The mechanisms of amelioration of glycemic control early after laparoscopic Roux-en-Y gastric bypass (LRYGB) or laparoscopic sleeve gastrectomy (LSG) are not fully understood.

**Methods:**

In this prospective, randomized 1-year trial, outcomes of LRYGB and LSG patients were compared, focusing on possibly responsible mechanisms. Twelve patients were randomized to LRYGB and 11 to LSG. These non-diabetic patients were investigated before and 1 week, 3 months, and 12 months after surgery. A standard test meal was given after an overnight fast, and blood samples were collected before, during, and after food intake for hormone profiles (cholecystokinin (CCK), ghrelin, glucagon-like peptide 1 (GLP-1), peptide YY (PYY)).

**Results:**

In both groups, body weight and BMI decreased markedly and comparably leading to an identical improvement of abnormal glycemic control (HOMA index). Post-surgery, patients had markedly increased postprandial plasma GLP-1 and PYY levels (*p* < 0.05) with ensuing improvement in glucose homeostasis. At 12 months, LRYGB ghrelin levels approached preoperative values. The postprandial, physiologic fluctuation returned, however, while LSG ghrelin levels were still markedly attenuated. One year postoperatively, CCK concentrations after test meals increased less in the LRYGB group than they did in the LSG group, with the latter showing significantly higher maximal CCK concentrations (*p* < 0.012 vs. LRYGB).

**Conclusions:**

Bypassing the foregut is not the only mechanism responsible for improved glucose homeostasis. The balance between foregut (ghrelin, CCK) and hindgut (GLP-1, PYY) hormones is a key to understanding the underlying mechanisms.

## Introduction

Bariatric surgery is the most successful weight loss therapy for morbid obesity, achieving markedly improved glycemic control (including diabetes resolution in most patients) and satiety hormone balance [1–3]. Roux-en-Y gastric bypass (RYGB) is effective for treating morbid obesity, inducing a marked and sustained weight loss, even extending to >10 years [1, 4–8]. Many surgeons therefore consider RYGB as the bariatric procedure of choice, and nowadays, a laparoscopic approach is most often undertaken (LRYGB) [1, 9–11].

Laparoscopic sleeve gastrectomy (LSG) is a newer approach, initially applied to superobese patients with severe co-morbidities where initial weight loss is intended to enable a later, definitive LRYGB or biliopancreatic diversion duodenal switch as part of a staged concept [12]. Recently published prospective studies compared results from a sole LSG operation to LRYGB [13–17]. Equal early weight loss, markedly improved glucose homeostasis, and increased insulin, glucagon-like peptide 1 (GLP-1), and peptide YY (PYY) levels after both procedures were reported, though LSG seemed easier to perform and associated with fewer complications [15, 17]. The mechanisms of weight loss following bariatric surgery include restriction, malabsorption, and humoral changes [3, 17–22]. Bypass procedures induce hormonal changes that ameliorate or even lead to complete remission of type 2 diabetes mellitus [3, 18–21]. In this present 1-year prospective, randomized trial, we compared LRYGB and LSG outcomes and investigated potential mechanisms responsible for the observed weight loss and metabolic effects, focusing on the main two hypotheses: foregut vs. hindgut theory [19, 20].

## Materials and Methods

### Patients

All studies were performed according to the principles of the Declaration of Helsinki. The Local Research and Ethics Committee in Basel approved the study. The included patients are a subgroup of an ongoing “Swiss Multicenter Bypass or Sleeve Study” (SM-BOSS, NCT00356213), comparing LSG and LRYGB with regard to primary endpoints, effectiveness and safety, to be closed as soon as 100 patients per group have been operated. In this present special group analysis, only non-diabetic patients from our center were consecutively included for logistic reasons. Table 1 provides baseline demographics of the present study. All patients were informed of the risks and benefits of each procedure and provided written, informed consent. Computer-generated random numbers in a sealed envelope determined the type of surgery (LRYGB or LSG). All operations were performed laparoscopically and by the same surgeon. The LRYGB technique included a small gastric pouch with a 25-mm circular pouch-jejunostomy to a 150-cm antecolic Roux limb and an exclusion of 50 cm of biliopancreatic limb. The LSG was done along a 35-F bougie from the angle of His to approximately 3–4 cm orally to the pylorus.

**Table 1:** Baseline demographics

Parameter	LRYGB (*n* = 12)	LSG (*n* = 11)	*p* value
Male/female	3/9	3/8	0.90
Age (years)	41.4 ± 10.1	35.2 ± 10.7	0.18
Fasting insulin (μU/mL)	30.0 ± 16.9	28.2 ± 16.5	0.79
Fasting glucose (mmol/L)	5.8 ± 0.7	5.7 ± 1.0	0.67
HOMA index	8.0 ± 5.1	7.5 ± 5.5	0.84
HbA1c (%)	5.7 ± 0.3	5.6 ± 0.6	0.72
Weight (kg)	133.3 ± 30.8	120.2 ± 19.8	0.23
BMI (kg/m^2^)	47.6 ± 6.8	44.7 ± 5.3	0.25

Data are presented as means ± SD. No significant differences between the two groups were detected

### Study Design

In this randomized, prospective, parallel group trial, all patients underwent complete evaluation before the respective bariatric operation and during follow-up, including medications, nutritional behavior, anthropometric and clinical parameters, and blood sampling for glucose, triglycerides, cholesterol, and other laboratory tests.

For meal studies, subjects were admitted to the Clinical Research Center before the operation and 1 week and 3 and 12 months after the operation. After fasting overnight (at least 10 h), an antecubital vein catheter was inserted for phlebotomy. After taking the fasting sample, a 424-kcal (1,775 kJ) liquid test meal containing 15 g carbohydrates, 25 g proteins, and 28 g fat was served to stimulate hormone release. Blood was drawn at the following times: −15, 0 (corresponding to commencing meal intake), 15, 30, 45, 60, 120, and 180 min. Samples (10 ml/withdrawal) were collected into EDTA tubes containing aprotinin at a final concentration of 500 KIU/mL of blood and a DPP-IV inhibitor; samples were immediately processed and kept on ice to retard peptide breakdown. After centrifugation at 4°C, plasma samples were kept frozen at −20°C until analysis.

### Hormones

The following hormones were investigated: cholecystokinin (CCK), GLP-1, PYY, insulin, and ghrelin. CCK concentrations were measured using a sensitive radioimmunoassay based on an antiserum that recognizes the sulfated tyrosine residue of all CCK molecules, but has little cross-reactivity with sulfated gastrin (<1%) and no cross-reaction with unrelated gastrointestinal peptides. Plasma samples were extracted with ethanol, and ^125^I-CCK-8 was used as a label. The lowest concentration currently measurable was 0.6 pmol/L plasma, using CCK-8 as a standard (details previously described [23]).

The lowest ghrelin level detectable by the commercially available kit (Linco Research Inc. St. Charles, 63304 MO, USA) used is 93 pg/mL/100 μL sample. At 1 ng/mL, the intra- and inter-assay coefficients of variation were 10.0% and 14.7%, respectively [15].

GLP-1 was measured with a commercially available ELISA kit (Linco Research Inc.); the assay used is highly specific for measuring active GLP-1 but does not detect other GLP-1 forms (e.g., 1–36 amide, 1–37, 9–36 amide, or 9–37). This assay sequentially (1) captures active GLP-1 with a monoclonal antibody (binds specifically to the N-terminal region of active GLP-1 molecules); (2) removes unbound materials; (3) binds an anti-GLP-1–alkaline phosphatase detection conjugate to immobilized GLP-1; (4) removes unbound conjugate; and (5) quantifies bound detection conjugate by adding methyl umbelliferyl phosphate which, with alkaline phosphatase, forms fluorescent umbelliferone. Since the degree of fluorescence is directly proportional to the active GLP-1 concentration, the latter is interpolated from a curve using reference standards of known active GLP-1 concentrations. The intra- and inter-assay variabilities were below 9% and 13%, respectively. The lowest level of GLP-1 currently detectable is 0.25 pmol/L (100 μL plasma sample) [24].

PYY was measured with a commercially available kit (Linco Research Inc.). The guinea pig antibody displays 100% cross-reactivity with human PYY1-36 and human PYY3-36, but not with human pancreatic polypeptide, NPY, leptin, or ghrelin). [^125^I]PYY, purified by HPLC (specific activity 302 μCi/μg), was used as a label. The lowest level of PYY currently detectable is 10 pg/mL (100 μL plasma sample). The intra- and inter-assay variabilities were below 9% and 9%, respectively [15].

The lowest level of insulin currently detectable with the commercial radioimmunoassay (Cisbio International, 30200 Bagnols, France) used is 4.6 μU/mL (100 μL sample). The intra- and inter-assay coefficients of variation were 12.2% and 9.0%, respectively [15]. Blood glucose concentrations were measured using a commercial hexokinase-glucose-6-phosphate-dihydrogenase method (Roche, 4070 Basel, Switzerland).

### Statistical Analysis

Data analysis was performed using the statistical software package, SPSS for Windows V. 15.0 (SPSS Inc., Chicago, IL 60606-6306, USA). Values are reported as means ± SEM. Descriptive statistics were used for demographic variables, such as age, weight, height, and BMI. Hormones were analyzed by calculating time courses, the area under the plasma concentration time profiles (AUC), and maximum plasma concentrations (Cmax). The general linear model procedure of repeated measures ANOVA using simple contrast was used to test for significant differences in longitudinal changes from baseline. To test for significant differences between the two treatment groups, the Student’s independent *t* test and the Bonferroni–Holm correction to adjust for multiplicity of testing were used. All tests were two-tailed, with *p* < 0.05 considered statistically significant.

## Results

### Clinical Characteristics

Twelve patients were randomized to LRYGB and 11 to LSG. All surgical procedures were successfully concluded laparoscopically with no conversion to open surgery. Table 1 provides demographics: Both groups had similar preoperative characteristics, including BMI, a clearly disturbed glucose homeostasis, expressed as elevated fasting glucose and insulin concentrations, and a highly pathological HOMA index, indicating insulin resistance. Normal values for the HOMA index were defined from values obtained in a test series of 60 healthy normal weight persons at our institution. All patients underwent a complete evaluation at 1 week, 3 months, and 1 year postsurgery.

### Weight Loss and Glycemic Control

**Fig. 1:**
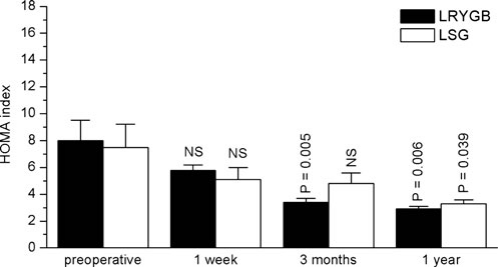
HOMA index in the two groups of patients (LRYGB and LSG) before, as well as 1 week and 3 and 12 months after the respective operation. Data are means±SEM

**Fig. 2:**
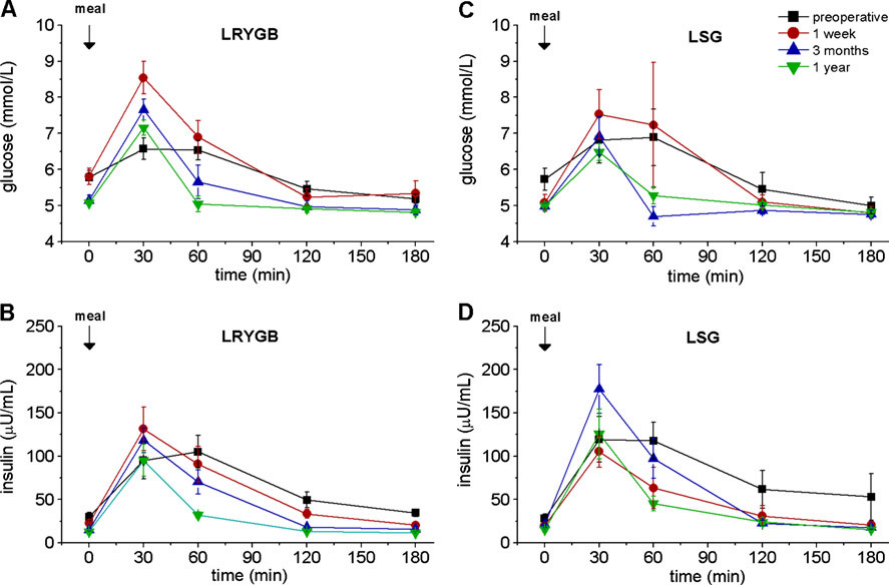
Fasting and meal-stimulated time courses of glucose and insulin in two groups of patients (LRYGB and LSG) before, as well as 1 week and 3 and 12 months after the respective operation. **a** Glucose in the LRYGB group, **b** insulin in the LRYGB group, **c** glucose in the LSG group, **d** insulin in the LSG group. Data are means ± SEM

Both procedures achieved a marked reduction in body weight and BMI (Table 2). LRYGB patients lost slightly more weight at 3 and 12 months, but this was not statistically significant. Accordingly, excessive BMI loss at 12 months was 77% (LRYGB) and 65.6% (LSG) (both *p* < 0.001 vs. preoperative values, no significant difference between the groups; Table 2). Fasting glucose and insulin levels dropped, and insulin resistance (HOMA index) returned to near normal values at 1 year, with no significant differences between the procedures (Figs. 1 and 2).

**Table 2:** Weight, BMI, and EBMIL (%) after LRYGB or LSG

Parameter	Treatment	Preoperative	1 week	3 months	1 year
Weight (kg)	LRYGB	133.3 ± 30.8	127.5 ± 29.8**	111.1 ± 24.3**	87.3 ± 24.1**
	LSG	120.2 ± 19.8	116.9 ± 18.7*	104.4 ± 17.7**	86.3 ± 15.6**
BMI (kg/m^2^)	LRYGB	47.6 ± 6.8	45.6 ± 6.9**	39.8 ± 5.8**	31.1 ± 7.5**
	LSG	44.7 ± 5.3	43.4 ± 5.2*	38.8 ± 4.8**	32.0 ± 5.0**
EBMIL (%)	LRYGB	0	9.8 ± 5.7**	35.8 ± 9.7**	77.0 ± 24.7**
	LSG	0	6.2 ± 5.7*	30.9 ± 9.6**	65.6 ± 21.2**

Data are presented as means ± SD. No significant differences between the two groups at the same time points were detected

*EBMIL* excessive BMI loss

*p ≤ 0.01; **p ≤ 0.001 = significant differences compared to preoperative within each group

### Meal-Stimulated Satiety Hormone Secretion

#### Upper Gastrointestinal Peptides

##### Ghrelin

**Fig. 3:**
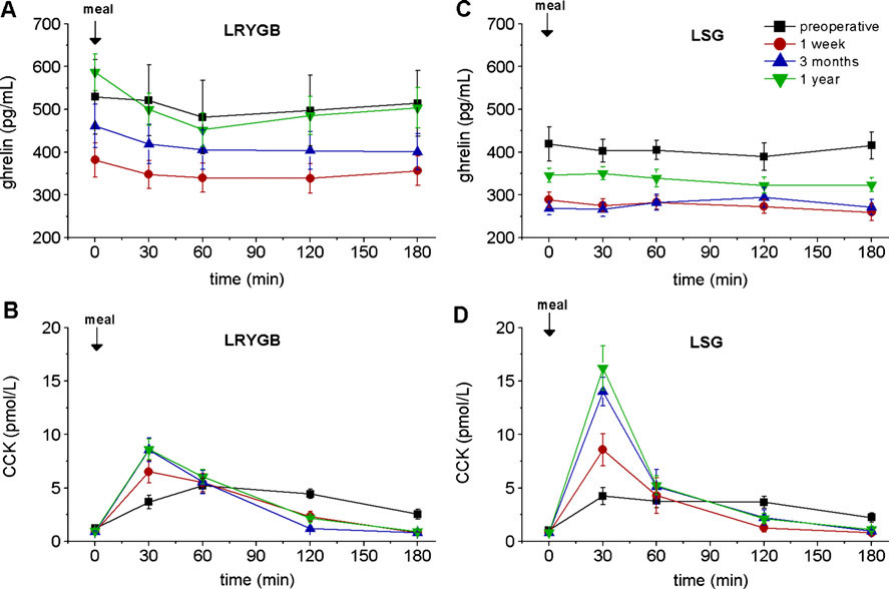
Fasting and meal-stimulated time courses of ghrelin and CCK in the two groups of patients (LRYGB and LSG) before, as well as 1 week and 3 and 12 months after the respective operation. **a** Ghrelin in the LRYGB group, **b** CCK in the LRYGB group, **c** ghrelin in the LSG group, **d** CCK in the LSG group. Data are means ± SEM. Details on the statistical analysis are given in Table 3

Physiological ghrelin levels in the non-obese are characterized by a rise during fasting periods and a rapid postprandial fall. The postprandial ghrelin inhibition was missing in all our patients preoperatively. One week postoperatively, ghrelin levels were lower than preoperatively in both groups (*p* < 0.05 vs. preop values). Lower ghrelin levels could be observed for fasting and meal-stimulated ghrelin levels as well. However, the decrease was more prominent in the LSG group, both at 1 week and 3 months postoperatively (lower AUC and lower Cmax, Fig. 3a, c; Table 3). Despite the initial fall, fasting ghrelin levels even exceeded preoperative values in the LRYGB group after 1 year, but the physiological response with the typical postprandial fall was reestablished. Contrarily, patients with LSG showed permanently attenuated ghrelin levels after 1 year (although slightly higher than at 3 months), interestingly without postprandial decline.

**Table 3:** Upper (ghrelin and CCK) and lower (GLP-1 and PPY) gastrointestinal peptides after LRYGB or LSG, preoperative and at 1 week, 3 months, and 1 year postsurgery

Parameter	Treatment	Preoperative	1 week	3 months	1 year
Ghrelin					
AUC (ng min/mL)	LRYGB	90 ± 15	62 ± 6*	74 ± 8	88 ± 8
	LSG	72 ± 5	49 ± 3***	50 ± 3***	60 ± 3
	*p* value	NS	NS	0.064	0.016
Cmax (pg/mL)	LRYGB	567 ± 86	395 ± 37**	469 ± 51*	587 ± 43
	LSG	451 ± 36	302 ± 18***	305 ± 22***	363 ± 17
	*p* value	NS	NS	0.048	0.001
CCK					
AUC (pmol min/L)	LRYGB	706 ± 80	622 ± 84	616 ± 77	701 ± 67
	LSG	599 ± 88	562 ± 122	824 ± 140*	897 ± 105**
	*p* value	NS	NS	NS	NS
Cmax (pmol/L)	LRYGB	5.6 ± 0.6	7.2 ± 1.0	8.7 ± 1.1**	8.7 ± 0.9**
	LSG	5.0 ± 0.7	9.3 ± 1.8	14.3 ± 1.4***	16.2 ± 2.1***
	*p* value	NS	NS	0.020	0.012
GLP-1					
AUC (pmol min/L)	LRYGB	326 ± 54	1,230 ± 172***	1,381 ± 183***	1,316 ± 302**
	LSG	297 ± 29	773 ± 140**	998 ± 160**	665 ± 125**
	*p* value	NS	NS	NS	NS
Cmax (pmol/L)	LRYGB	3.4 ± 0.8	15.1 ± 2.1***	21.2 ± 3.5***	21.2 ± 5.7*
	LSG	2.4 ± 0.4	10.0 ± 1.9**	14.3 ± 1.9***	12.0 ± 2.2**
	*p* value	NS	NS	NS	NS
PYY					
AUC (ng min/mL)	LRYGB	27.6 ± 2.8	53.5 ± 2.9***	43.1 ± 4.1**	38.3 ± 4.3*
	LSG	23.6 ± 1.4	48.7 ± 5.9**	35.2 ± 3.5**	31.7 ± 4.0
	*p* value	NS	NS	NS	NS
Cmax (pg/mL)	LRYGB	175 ± 18	389 ± 26***	320 ± 29***	280 ± 32**
	LSG	152 ± 10	353 ± 52**	270 ± 24**	230 ± 32**
	*p* value	NS	NS	NS	NS

Data represent means ± SEM. *p* < 0.05 = significant differences between the two groups at the same time points

*NS* not significant, *AUC* area under the concentration time profile, *Cmax* maximum plasma concentrations

*p ≤ 0.05; **p ≤ 0.01; ***p ≤ 0.001 = significant differences compared to the preoperative within group

##### CCK

Both groups showed a normal CCK response after stimulation with the standardized test meal before operation. Postoperatively, patients with LSG as well as with LRYGB had elevated CCK concentrations. The1-week values were markedly increased, but the effect was short-lasting (60 min). The postprandial test meal response 1 year postoperatively revealed a numerically smaller increase in the LRYGB group than that in the LSG group. Differences in maximal CCK concentrations were statistically significant (*p* < 0.012) (Fig. 3b, d; Table 3).

#### Lower Gastrointestinal Peptides

##### GLP-1

**Fig. 4:**
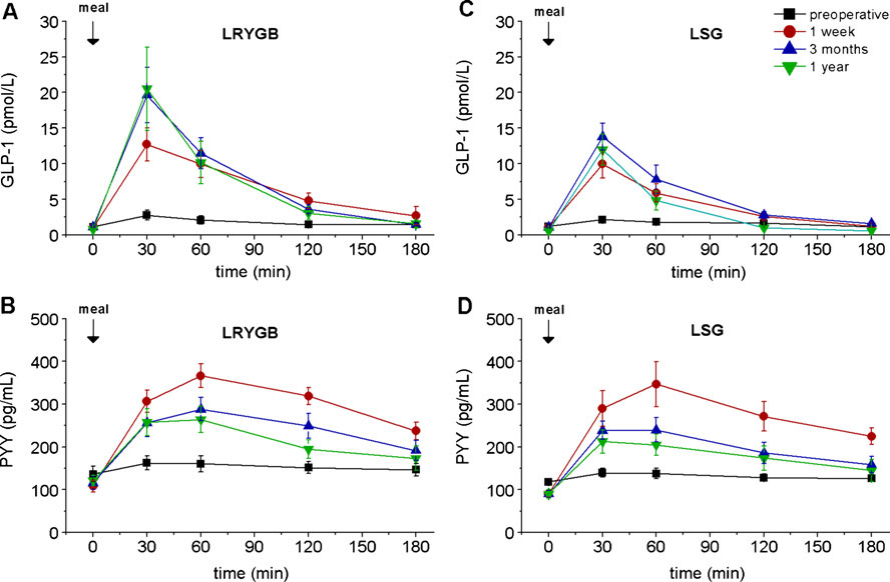
Fasting and meal-stimulated time courses of GLP-1 and PYY in the two groups of patients (LRYGB and LSG) before, as well as 1 week and 3 and 12 months after the respective operation. **a** GLP-1 in the LRYGB group, **b** PYY in the LRYGB group, **c** GLP-1 in the LSG group, **d** PYY in the LSG group. Data are means ± SEM. Details on the statistical analysis are given in Table 3

Both groups had a defective GLP-1 response to test meal intake before the operation. LRYGB patients exhibited an early marked increase in postprandial GLP-1 levels at 1 week after this form of bariatric surgery (*p* < 0.001 vs. preop; Fig. 4a, c; Table 3). The markedly increased GLP-1 response was unchanged after 3 months and 1 year in the LRYGB group; a similar but less prominent pattern was seen in the LSG group, although the AUC was numerically clearly smaller in the LSG patients.

##### Peptide YY

Before surgery, PYY levels did not significantly increase in response to food, suggesting a defective PYY response. Fasting PYY levels decreased after surgery in both study groups and expressed an exaggerated postprandial PYY response 1 week after the operation, an effect that was slightly less prominent but still present 3 months and 1 year later (Fig. 4b, d; Table 3). The response pattern and secretory output were comparable, with no significant differences.

## Discussion

Bariatric surgery is the only current treatment option that leads to sustained weight loss and reduction in mortality for morbidly obese patients [1–3]. LRYGB is presently the gold standard, resulting in greater weight loss than purely restrictive procedures [1, 2]. LSG is a novel bariatric procedure that avoids intestinal bypass. In this prospective, randomized, controlled study, we show that, at 1 year, weight loss and sustained improvement in glycemic control manifest themselves to almost the same extent and at a similar pace after LRYGB and LSG procedures, which are both safe and highly effective therapies for morbid obesity. These outcomes contrast with those following adjustable gastric banding, a purely restrictive procedure [23]. We conclude that LSG might be a reliable alternative to LRYGB and also suggest that foregut exclusion cannot be the sole explanation for the marked weight loss and improvement in glucose metabolism [19–21, 23].

One major focus of this investigation was the time courses of satiety hormones based on their release site (foregut, hindgut). The general consensus is that many of the improvements in glycemic control achieved by bariatric surgery are likely to be associated with alterations in the secretion of hormones from the gut. LSG restricts the volume capacity of the stomach; in contrast, the LRYGB procedure excludes food from the stomach and the proximal small intestine, thereby exposing the distal gut to altered chyme [3, 18, 21]. It is most likely that both duodenal exclusion (foregut hypothesis) and rapid exposure of distal small intestine to nutrients (hindgut hypothesis) are mechanisms that potentially contribute to improved glycemic control. The two procedures induce slightly different hormonal patterns over time, although the improvement in weight loss, BMI, and glucose homeostasis remains comparable 1 year after surgery. Our observations provide a basis for explaining the procedures’ possible mechanisms.

### Foregut vs. Hindgut Hypotheses

It has been proposed that the anatomical rearrangement alters food passage dynamics, evoking changes in gut hormone secretion to food [3, 19–21, 23]. A variety of studies focusing on hormonal changes after LRYGB have demonstrated decreased ghrelin levels on the one hand and increased levels of GLP-1, PYY3-36, and adiponectin after weight loss on the other hand. These changes support the possibility of hormonal weight-independent effects [3, 18–21, 23, 24]. Very few studies have investigated hormonal changes after LSG [14, 15]; here, we document markedly decreased ghrelin levels and increased concentrations of CCK, GLP-1, and PYY3-36.

Consistent with the hindgut hypothesis, RYGB creates a shortcut to the distal small intestine; the stomach is restricted, and chyme is excluded from the foregut. Endogenous GLP-1 and PYY levels were low in both groups before surgery, suggesting diminished endogenous levels of these hormones in obese individuals [3, 18, 21]. Here, postprandial GLP-1 and PYY values dramatically and lastingly increased after RYGB, most likely by direct nutrient contact with distal intestinal L cells. Surprisingly, as this procedure does not alter small intestine food passage, a similar increase in GLP-1 and PYY was seen after LSG. An explanation for this might be that GLP-1 release is not only triggered via direct nutrient contact with distal L cells [25]. Another stimulus for GLP-1 secretion is derived by proximal nutrient signals, e.g., increased CCK secretion. CCK blood levels are stimulated by long chain free fatty acid formation [26]. This could explain the markedly more pronounced CCK stimulation in the LSG group. As expected, both operations greatly enhanced postprandial GLP-1 and PYY surges. The hormonal changes and weight loss clearly enhanced insulin secretion and sensitivity, as estimated by the HOMA index. Of note, glucose homeostasis began improving 1 week after surgery, even before any meaningful weight loss. Although weight loss is associated with improved glucose control, it has been suggested that post-RYGB improvement in glucose metabolism is greater than with equivalent weight loss from other regimens [3, 5–8, 21, 27]. Here, we provide experimental evidence that improvement in glucose control is similar with LSG as well as with LRYGB.

Furthermore, at 1 year follow-up, BMI values were similar with both procedures, and the changes paralleled improvements in dyslipidemia (lower triglycerides, increased HDL cholesterol levels) [28]. The LSG results are interesting: In contrast to adjustable gastric banding, which like LSG is a gastric restrictive procedure, ameliorated glycemic control was observed already at 1 week postoperatively, even before substantial weight loss occurred. These findings concur with results from Lee et al., who compared matching patients with moderate obesity (BMI 27–35 kg/m^2^) but poorly controlled diabetes undergoing either sleeve gastrectomy or a sleeved version of RYGB. After 6 months, both procedures achieved equivalent weight loss and improvement in glycemic control [29, 30].

A potential explanation for PYY and GLP-1 increases following LSG could be accelerated gastric emptying and earlier contact of chyme with the L cells of the hindgut. Scintigraphic studies performed up to 2 years after LSG showed accelerated gastric emptying for solid and liquid foods [31, 32]. In contrast, a recent MRI study, focused on the motility changes postoperatively, could demonstrate two different functional regions in the remnant stomach: The antral motility remained unchanged even very early postoperatively, whereas the sleeved stomach seemed to be nearly aperistaltic even 6–8 months postoperatively [33].

The clinical importance of increased PYY levels is unclear: over-expression of PYY in transgenic mice did not change weight or food intake, but other studies have proposed that pharmacologic concentrations of PYY function as anorexic signals, reducing food intake, body weight, and body fat mass [34, 35]. In summary, glycemic control improved in both groups equally over a 1-year follow-up period, associated with marked increases in GLP-1 (although slightly more so in the RYGB group) and PYY secretions and changes in meal-stimulated CCK release.

### Ghrelin Hypothesis

The relationship between bariatric surgery and ghrelin levels is controversial. Some years ago, Cummings and co-workers provided initial evidence for reduced secretion of the orexigenic, prodiabetic, foregut hormone, ghrelin, contributing to the anorexic and antidiabetic effects of RYGB [36]. Other authors have failed to confirm these findings [37]. Here, we present postprandial ghrelin profiles over a 1-year period following both procedures. The first interesting observation is the lack of postprandial suppression of ghrelin levels before surgery in both groups, suggesting that the physiological regulation of ghrelin secretion is, at least, partially lost in morbidly obese subjects. In sleeve gastrectomy patients, ghrelin levels were markedly reduced and remained extremely low for several months after the operation, but showed a small (but still markedly reduced) increase in ghrelin after 1 year. This finding is not surprising because the stomach produces the majority of ghrelin. In contrast, ghrelin levels were reduced in post-RYGB patients, but not as pronouncedly so as with LSG. Over time, ghrelin levels returned to preoperative levels but with a major difference: Patients had regained the physiologic postprandial suppression after meal ingestion 1 year after surgery.

The changes in ghrelin can contribute to the marked decrease in weight loss, appetite, and food intake that follows both surgical procedures, but should be more prominent after the LSG procedure [7, 36]. Recent studies in mice deficient for both ghrelin and its receptor indicate that a complete functional absence of ghrelin signaling is sufficient to decrease body weight and fat mass [38–40]. The changes in ghrelin should also contribute to improved glucose homeostasis, as ghrelin can stimulate insulin counter-regulatory hormones, suppress the insulin-sensitizing adipokine, adiponectin, and inhibit insulin secretion [28, 38–40]. From these results, we infer that part of the glycemic improvement after LSG arises from reduced ghrelin secretion.

Our observations are intriguing and to a certain extent unexpected as neither the upper (foregut) nor the distal (hindgut) intestinal hypothesis can fully account for improvement in glucose homeostasis. These results suggest rather that the balance between foregut (ghrelin, CCK) and hindgut hormones (GLP-1, PYY) is a key for understanding the improved glucose homeostasis. Another important factor for the improvement of glucose homeostasis is nutrient sensing and metabolism influencing insulin sensitivity, thus supporting the nutrient-related hormone release and balance. To completely understand the effects and interactions how different bariatric procedures influence nutrient-sensing regulatory mechanisms, influence gut hormone balance, and finally affect glucose homeostasis, more research is warranted.
